# Effect of *Astragalus membranaceus* Oral Solution on Lifespan and Learning and Memory Ability of Honey Bees

**DOI:** 10.1155/2020/5745048

**Published:** 2020-04-11

**Authors:** Tao Hong, Long-Xue Li, Xiao-ping Han, Jing-liang Shi, Cai-yun Dan, Zhi-Yong Liu, Xiao-Bo Wu

**Affiliations:** ^1^Experimental Animal Center, Jiangxi University of Traditional Chinese Medicine, Nanchang 330004, China; ^2^Honeybee Research Institute, Jiangxi Agricultural University, Nanchang 330006, China; ^3^Key Laboratory of Pharmacology of Traditional Chinese Medicine in Jiangxi, Nanchang 330004, China

## Abstract

In this study, the effects of *Astragalus membranaceus* oral solution on lifespan and learning and memory abilities of honey bees were evaluated. Two groups of bees were fed with sucrose syrup (50%) containing low dose (1.33%) and high dose (13.3%) of *A. membranaceus* oral solution, respectively. The proboscis extension response (PER) analysis was applied to examine the learning and memory capabilities of bees. Two genes related to memory formation in honey bees were determined by real-time PCR. High dose (13.3%) of *A. membranaceus* significantly decreased the mean lifespan of bees compared to the bees fed with low dose (1.33%) and control bees. No significant differences in lifespan of bees were found between low-dose-fed bees and control bees. The results of PER experiments showed apparent improvement in the memorizing ability of the high-dose group (in comparison with the control group). Moreover, the relative expression levels of *Nmdar1* in the low-dose group and control group were significantly lower than those in the high-dose group. It is preliminarily concluded that *A. membranaceus* has an adverse effect on the mean lifespan of honey bees but might be helpful in strengthening memories.

## 1. Introduction

*Astragalus membranaceus* (Huang-Qi) is a well-known tonic herbal medicine [1]. In China and Southeast Asia, it has been widely applied for cancer and immune disorder treatments for thousands of years [2–6]. It is also used in Asian cuisine because of its rich nutritional values [7]. *A. membranaceus* contains seventeen kinds of amino acids and various trace elements necessary for the human body [8–11].

As a favorite Chinese medicine for drug or food, *A. membranaceus* safety has also attracted researchers' attention. In the past, mammals like rats and mice are the most commonly selected animal models for long-term toxicity experiments with a period from 90 days up to half a year. Nowadays, some toxicologists began to explore the use of invertebrates as an alternative for mammals for long-term studies. Researchers have found that *Huang-Qi* could speed up the growths and increase the body weights of silkworms; however, it would reduce the in vitro secretion of silk protein, thus shortening the lifespan of silkworm moth [12].

Honey bees are emerging model insects for biomedical research with the advantages of moderate size, tractable large-scale breeding, complex and precise social structure, complete genomic information, and short lifespan [13–15]. The short life cycles of the bees are particularly advantageous for observing the long-term pharmaceutical toxicity effects of various food or chemicals. At the same time, the learning and memorizing ability of bees can be used as an important indicator for the healthy development of bee colonies and their foraging efficiency [16–20]. Among them, related gene expressions of the glutamate receptor A (GluRA) and the N-methyl-d-as-partic acid receptor (Nmdar) were important [21, 22]. The Nmdar1 receptor is a very important excitatory amino acid receptor in the learning and memory process of biological individuals. *GluRA* is considered as the main excitatory neurotransmitter in the regulation of learning and memory behaviors of the vertebral brains, which plays a key role in cell differentiation and synapse formation during the development of nervous system. It is a vital neurotransmitter in the process of bee learning and memory, thus has a strong correlation with the learning and memory abilities of bees.

The short life cycle of bees makes it convenient to observe *A. membranaceus* oral solution effects on viability of this species. The importance of the learning and memory capabilities of bees leads to the consideration of whether *A. membranaceus* solution would affect the bees in these aspects. To better understand the toxicity of the oral solution of *A. membranaceus*, the aim of this study was to investigate the impact of high (13.3%) and low (1.33%) doses of *A. membranaceus* solution on the longevity and memory-related traits of *A. mellifera* worker bees.

## 2. Materials and Methods

### 2.1. Experimental Setup and Handing of Bees

Experiments were conducted during September-October 2018 at Honeybee Research Institute of Jiangxi Agricultural University, Nanchang (28.46°N, 115.49°E), China. The colonies of honey bees (*Apis mellifera*) were managed with standard beekeeping practices, and handling of bees in the laboratory was performed according to the standard protocol of William et al. [23]. Three *A. mellifera* colonies were chosen to produce the frames of sealed brood for the whole experiments. When individual bees on the frame were coming out of the cells, we then removed the frame from each colony which were not exposed to *A. membranaceus* and transferred into an incubator in the dark at 35°C with 75 ± 5% relative humidity. Newly emerged worker bees were collected in 24 h. Bees from each colony were separated into 3 groups in wooden cages (14 × 12 × 16 cm). 50% sucrose water containing low dose (1.33%) and high dose (13.3%) of *Astragalus membranaceus* oral solution was fed to the 2 groups, respectively, and control bees were fed only 50% sucrose water. The experiment was repeated three times with bees from three different colonies. During this time (keep feeding a syrup containing *A. membranaceus* until all bees die), the caged honey bees were fed with abundant syrup (containing *A. membranaceus*) from a 50 mL centrifuge tube through a hole on the cage [24, 25].

### 2.2. Chemical Reagents and Apparatus

Different reagents used in this study were *A. membranaceus* solution (Huangqi jing, catalogue number: Z32020370, Yangzijiang Pharmaceutical Group), sucrose (catalogue number: Xiqiao Chemicals, Co., Ltd.), Oligo (American Yingjie Life Technology) and Rox Reference DyeII (TakaRa), RNA extraction kit (Beijing Quanshijin Biotechnology, Co., Ltd.), dTNP mixture reverse transcriptase M-MLV (200 U/*μ*L), fluorescent dye SYBRR Premix Ex Taq™ II, and Rox Reference DyeII (TaKaRa).

Apparatuses used in this study were 7500 software v2.3 real-time PCR instrument, Eppendorf Mastercycler personal, NanoDrop 2000 spectrophotometer (Thermo Scientific, Wilmington, DE, USA), GZ-250-GSI biochemical incubator (Shaoguan Guangzhi Technology Equipment Development, Co., Ltd.), FA2004N purification workbench (Shanghai Puchun Technology, Co., Ltd), and GAFFA tape (Ningbo Jixiang Plastic Science & Technology, Co., Ltd).

### 2.3. Longevity of Worker Bees

Three groups of bees (control: *N* = 324; low dose: *N* = 346; high dose: *N* = 309) were placed in a biochemical incubator (T: 35°C; RH: 75 ± 5%) for rearing and were fed with the corresponding solutions of pure sucrose; 1.33% and 13.30% *A. membranaceus* syrups were fed with sufficient amounts (~5 mL solution/100 bees). Bees have received adequate syrup supply each day every morning and records of mortality were kept in each group until all of the bees died (when we touch all parts of the bee's body, there is no response and it is in a rigid state, we considered that the bee is dead).

### 2.4. Behavioral Analysis of Honey Bees

PER (proboscis extension response) behavioral experiments were performed to detect the learning and memory abilities of 7-day-old worker bees. 90 worker bees of each group were taken (with tweezers) from the rearing cages. These worker bees were subjected to a short-freeze treatment with ice cubes (3-5 min), and a previous study has showed that ice chilling did not affect the memory ability of bees compared with other cold immobilization methods [26]. After bees were immobilized on ice, each bee wash harness into a U-shaped metal tube with thin strips of GAFFA tape (DUCT tape/GAFFA REPAIR tape) so that the whole body was captured in the tube, while the head and two prolegs were free. Then, bees were transferred into a biochemical incubator (T: 35°C; RH: 75 ± 5%) to recover under a dark condition. After more than 2 hours of starvation, the harnessed bees on the metal tube were taken out of the incubator, and the inactive bees (after touching their head and antenna) were discarded, and then the learning and memory abilities of the remaining healthy worker bees were studied [27, 28]. Firstly, we used two scents, limonene and vanilla, as positive and negative unconditioned stimulus, respectively [29]. Bees were trained that limonene plus 1 M (1 mol/L) sugar solution was the reward, while vanilla plus saturated salt solution was the punishment (fear learning). We counted the learning success rate of bees in three repetitions of training, and three repetitive trainings with an interval of 6 min were given using above two scents. Secondly, bees with successful learning in three repetitive training were fed enough sugar (2-3 drops) and transferred into an incubator for overnight storage. Then, the memory test was performed in the second morning with three times test for each bee using the above two scents, an interval of 6 min was set between the three times testing. We considered PER success of a bee if it made more correct responses to the positive and negative stimuli than incorrect responses [28].

### 2.5. Effect of *A. membranaceus* on the Expression of Learning and Memory Related Genes in *Apis mellifera* Honey Bees

#### 2.5.1. Sample Collection

The worker bees (7 days old) were taken from the rearing cages for exploring the differences of learning and memory-related genes expression among three groups. A total of nine bees involved three group samples were sampled into EP tubes under liquid nitrogen freezing condition in each group. After 5 min, bees in tubes were stored at -80°C for subsequent qPCR experiments.

#### 2.5.2. RNA Extraction and Reverse Transcription for cDNA

Heads of three bees were pooled as a single sample for RNA extraction. The total RNA extraction using an RNA extraction kit (Beijing Quanshijin Biotechnology Co., Ltd.) according to the manufacturer's protocol. For genomic DNA removal, an on-column DNase digestion with the RNase-free DNase set (Beijing Quanshijin) was carried out according to the manufacturer's instructions. RNA was eluted using 40 *μ*L RNase-free water and stored at −80°C [30]. NanoDrop 2000 spectrophotometer (Thermo Scientific, Wilmington, DE, USA) was used to check the RNA quality. Values between 1.8 and 2.0 were considered acceptable as pure RNA.

#### 2.5.3. Reverse Transcription of RNA

First-strand cDNA synthesis was performed by QuantiTect Reverse Transcription Kit (Beijing Quanshijin Biotechnology, Co., Ltd) in accordance with the manufacturer's instructions. The cDNA was kept at −20°C until use. The quality of cDNA was measured by a NanoDrop 2000 spectrophotometer (Thermo Scientific, Wilmington, DE, USA).

#### 2.5.4. Design of Fluorescent Quantitative PCR Primers and Real-Time PCR

Primers were designed for two memory-related genes (*Nmdar1* and *GluRA*) of honey bee in GeneBank; Primer 5.0 was used to design the primer sequence of the target gene according to the principle of fluorescent quantitative primer design [31]. The internal reference gene (GAPDH) was adopted from a previous study [30]. The primer sequences and reaction systems are shown in Tables 1 and 2.

The RT-qPCRs were performed on an Applied Biosystems ABI 7500 machine. The PCR reaction mixture (10 *μ*L) contained 1 *μ*L cDNA, 5 *μ*L SYBR® Premix ExTaq™ II, 0.2 *μ*L Rox Reference Dye, 0.4 *μ*L each of the forward and reverse primers, and 3 *μ*L ultra-pure sterile water. The reaction thermal profile included an initial denaturation (95°C 30s), quantification for 40 cycles (95°C 10s, 60°C 1 min), and a dissociation from 50°C to 90°C (elevated by 1°C every 6 s). When the fluorescent quantitative PCR machine ran out, the error of three technical replicates' values of each reaction was validated within 0.5. The dissolution curve was established to collect the Ct values of the target gene and the internal reference gene (Wang, 2014); and finally, the relative expression of each gene was calculated following the method by Huang et al. [24].

### 2.6. Data Analysis

The statistical analysis software SPSS 22.0 was used to test the longevity of the three groups of bees. The PER and gene expression results were further analyzed using ANOVA and multiple ANOVA. When *P* < 0.05, the LSD test was used to determine the difference between the groups.

## 3. Results

### 3.1. Effect of *A. membranaceus* Solution on the Average Lifespan of *A. mellifera* Honey Bees

**Figure 1:**
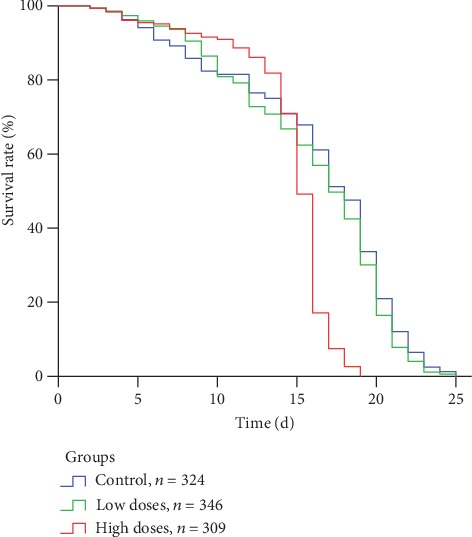
Effect of *Astragalus membranaceus* oral solution on the survival rate of *Apis mellifera* workers. Bees were fed with different concentrations of *Astragalus membranaceus* oral solution (13.3%, 1.33%, or 0%) in three groups corresponding to three colors, respectively. Bees fed with 0% *Astragalus membranaceus* oral solution were used as control group. Different letters in the column of average lifespan indicate significant difference (*P* < 0.05). *N* represents the sample size marked on the trend line.

**Figure 2:**
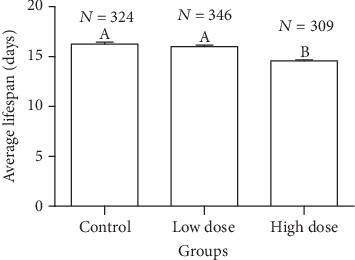
Effects of *Astragalus membranaceus* oral solution on the lifespan of *Apis mellifera* worker bees. Different letters in the column of average lifespan indicate significant difference (*P* < 0.05). *N* represents the sample size marked at the top of column.

Figure 1 shows that the survival ability of worker bees fed with high dose of *A. membranaceus* solution after 15 days of age was significantly lower than that of the other dose groups, while the survival ability of the control group and the low dose group was not significant. The results in Figure 2 show that the average lifespan of bees in the high-dose group was significantly lower than that in the low-dose group and the control group (*X*2 = 147.987, *P* < 0.001). Lifespan of worker bees in low-dose and control groups are significantly at par with each other (LSD: *P* = 0.233).

### 3.2. Effects of *A. membranaceus* on the Learning and Memory Abilities of Worker Bees

**Figure 3:**
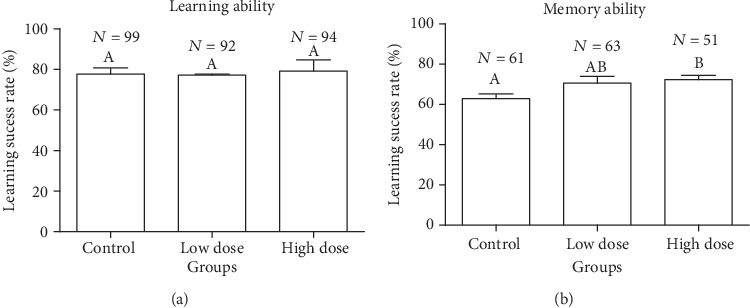
Effects of different concentrations of *Astragalus membranaceus* oral solution on the success rate of PER (a, b). The histogram of learning ability shows the percentage of bees that successfully passed the learning conditioning, and the histogram of memory ability shows the percentage of bees that achieved correct score in the PER memory test. Each group has a single error bar, which indicates the mean ± SE of three biological replicates. Different letters above the bars indicate significant difference (*P* < 0.05). Columns in the each graph sharing the common letters are not significantly different from each other. *N* represents the sample size.

Figure 3(a) illustrates the insignificant effect of *A. membranaceus* on the learning ability of western honeybees (*F*2, 6 = 0.082, *P* = 0.923). The learning performance of low dose and high dose of *A. membranaceus*-treated group of bees showed little deviation from that of the control group. As shown in Figure 3(b), the memorizing ability of bees treated with low dose of *A. membranaceus* were not significantly different from that of the control group (LSD: *P* = 0.086), but the memory capacity of the high-dose group was significantly higher than that of the control group (LSD: *P* = 0.044). There was no significant difference in memorizing ability between the low-dose group worker bees (LSD: *P* = 0.644). It is preliminarily concluded that the high dose of *A. membranaceus* solution can promote the memory strength of worker bees.

### 3.3. Effect of *A. membranaceus* on the Relative Expression of Learning and Memory-Related Genes in *A. mellifera* Honey Bees

**Figure 4:**
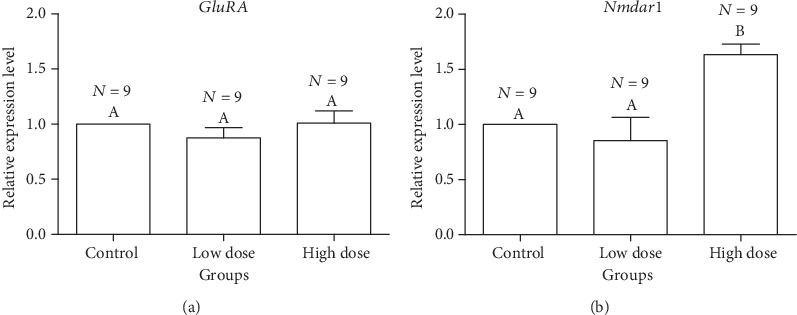
Effects of different concentrations of *Astragalus membranaceus* oral solution on the relative expression of genes GluRA (a) and Nmdar1 (b) of *Apis mellifera* workers using RT-qPCR. Each error bar below the letter indicates the mean ± SE of three biological replicates. Columns in the each graph sharing the common letters are not significantly different from each other.

Figure 4(b) reveals that expression of the *Nmdar1* gene in the control group was not significantly different from that in the low-dose group (LSD: *P* = 0.486), while the relative expression of the *Nmdar1* gene in the high-dose group was significantly higher than the other two (*F*2, 24 = 6.877, *P* = 0.037); the relative expression of the GluRA gene (Figure 4(a)) in the high (13.3%) and low (1.33%) doses of A*. membranaceus* solution showed no significant difference from that in the control group (*F*2, 24 = 0.790, *P* = 0.496).

## 4. Discussion

*A. membranaceus*, one of the traditional Chinese medicines, has been recognized as typical representatives of complementary and alternative medicines [32]. However, toxicity (or side effects) of long-term use of *A. membranaceus* is unknown. Yang et al. [34] studied the long-term (three months or 90 days) toxicity of Huang-Qi lycopene powder on rats and dogs [33]. The results showed that the nontoxic dose of Huang-Qi to rats was 39.9 g/kg, which is 70 times higher than the usual dosage used by human adults (60 kg BW); the nontoxic dose to dogs is 19.95 g/kg, equivalents to 35 times of the adult dose. Yang has reported that Huang-Qi decoction showed no obvious toxicity towards rats [34]. Polysaccharides are one of the main components of Huang-Qi. To examine on the toxicity of Huang-Qi polysaccharides, Wang has targeted on the acute toxicity and mutagenicity of these chemicals while also conducted experiments with 30 d feeding test method [35]. The results suggested that Huang-Qi polysaccharides are nontoxic, with both mutagenic test and 30 d feeding test resulting negative. From the above research, we found that using mammals to observe for the possible side effects of long-term use of *A. membranaceus* is not sufficiently convincing, since the experimental cycles of 90 to 180 days do not cover the entire lifespan of the animals. Use of bees as model makes it easier to observe the effects of *A. membranaceus* on the entire lifespan. Our findings showed that high dose (13.30%) of *A. membranaceus* significantly shorten the lifespan and survival of honey bees as compared to low dose (1.33%) of *A. membranaceus* and control bees. The cause of this phenomenon is speculated that it may be the toxic effect of *A. membranaceus*, because the dose used in this experiment is directly converted based on human clinical dosage (Editorial Committee of Zhonghua Bencao National Traditional Chinese Herb Administration, 1999).

Our study found that with increasing proportion of *A. membranaceus* in the food, the PER success rate increased. The PER experiment exhibited no difference is found in learning, and one difference for high dose found in memory. This indicated that *A. membranaceus* solution has no effect on the learning ability of western honey bee worker bees, whilst a higher dose of *Huang-Qi* can probably enhance the memory ability of bees. Actually, the learning ability of the bees during the training process was more dependent on the short-term memory (within 6 min), it is also influenced by the physiological state of the bees. In this experiment, the memory test of bees was mainly aimed at long-term memory (>12 h). Specifically, above results indicate that *A. membranaceus* can improve the long-term memory of bees to a certain extent but has no significant effect on short-term memory.

Some previous studies investigated the regulation of the expression of *Nmdar1* and gene [28, 36, 37]. These two genes that are closely related to the learning and memory of bees to observe the effects of *A. membranaceus.* The results of qPCR test showed that the relative expression of *Nmdar1* gene in high-dose group was significantly higher than that in the other two groups, while the expression level in low-dose group was slightly lower than that in control group. The relative expression of *GluRA* gene did not alter a lot from the control group to the treated experimental groups. The difference between the two experimental groups was not significant, and both of them showed that the relative expression of the two genes in the low-dose group decreased compared with the control group and the high-dose group. Whether the specific dose of *A. membranaceus* solution could have boosting effects on the learning and memorizing abilities of bees requires analysis from further studies.

## 5. Conclusions

In this study, honey bees were used as experimental animal model, with their lifespan and learning and memory abilities analyzed to test on the toxicity effects of *A. membranaceus* oral solution. It is preliminarily concluded that *A. membranaceus* has an adverse effect on the average lifespan of honey bees but might be helpful in improving memory strength.

## Figures and Tables

**Table 1:** Primer sequences of genes were descripted for RT-qPCR.

Gene	GeneBank accession number	Forward primer (5′-3′)	Reverse primer (5′-3′)
*Nmdar1*	NM_001011573.1	ACTGACGGTACCGAAGAGGA	CCCATACCATGCCCAACACT
*GluRA*	XM_395227.4	GGATGAAAGAAGGAAAAGGATA	ACAGTAACAATAACAACAGCGAT
*GAPDH*	XM_393605	GATGCACCCATGTTTGTTTG	TTTGCAGAAGGTGCATCAAC

**Table 2:** Reaction system of quantitative PCR assays.

Reagent	Volume (*μ*L)
cDNA	1.0
ddH2O	3.0
S YBR GREEN II	5.0
Forward primer	0.4
Reverse primer	0.4
ROX correction fluid	0.2
Total volume	10.0

## Data Availability

The data used to support the findings of this study are available from the corresponding author upon request.
